# A Low-Complexity Hybrid Handover Strategy for LEO NTN: Balancing Stability and Link Quality

**DOI:** 10.3390/s26051449

**Published:** 2026-02-26

**Authors:** Khalid Aldubaikhy

**Affiliations:** Department of Electrical Engineering, College of Engineering, Qassim University, Buraidah 51452, Saudi Arabia; k.aldubaikhy@qu.edu.sa

**Keywords:** handover management, LEO satellite networks, non-terrestrial networks (NTN), mega-constellation, ping-pong effect, multi-attribute decision making, Starlink, connection stability

## Abstract

The deployment of low Earth orbit (LEO) satellite mega-constellations enables global broadband access, but their high orbital velocity demands frequent handover decisions that critically impact service continuity. Conventional strategies that maximize instantaneous signal quality often trigger excessive handovers, while stability-focused approaches may sacrifice link performance. In this paper, we propose the Hybrid Handover Strategy (HHS), a low-complexity algorithm that addresses this trade-off. The HHS utilizes a multi-attribute utility function that integrates the signal-to-interference-plus-noise ratio (SINR), satellite elevation angle, and network load with a novel logistic-decay stability bonus mechanism. We provide a formal mathematical analysis of the algorithm’s stability and performance trade-offs. To ensure industrial relevance, the strategy is validated using a high-fidelity simulator driven by real-world two-line element (TLE) data from the Starlink constellation. Results demonstrate that the HHS reduces the handover frequency by 64% compared to SINR-based benchmarks while maintaining service availability of 90.2%. The proposed algorithm delivers these improvements with significantly smaller computational overhead than machine learning approaches, making it suitable for resource-constrained on-board processing and ground terminals.

## 1. Introduction

Sixth-generation (6G) wireless systems are expected to integrate terrestrial and non-terrestrial network (NTN) infrastructures [1]. Within this paradigm, constellations of low Earth orbit (LEO) satellites play a pivotal role in realizing the vision of ubiquitous global connectivity. These orbital networks can support high-throughput, low-latency broadband services in underserved regions and enable large-scale Internet of Things (IoT) deployments. For example, a stationary user terminal in a representative urban location (e.g., Riyadh) could see dozens of satellite handovers in just a few hours, because visible LEO satellites move quickly in their orbits.

However, the same orbital dynamics that make LEO constellations work with low latency also make it very challenging to manage mobility. Unlike geostationary satellites, LEO satellites move quickly relative to the ground, which means that they can only be seen for short periods of time. This makes it necessary to frequently and reliably switch between satellites. This process is a very important factor in the end-user’s quality of service (QoS) [2]. This challenge creates a trade-off: strategies that maximize instantaneous link quality often trigger excessive handovers, while stability-focused strategies may maintain weaker connections. For instance, always selecting the satellite with the highest signal-to-noise ratio (SNR) can lead to frequent handovers and the well-documented ‘ping-pong’ effect [3,4]. This problem is further complicated by the dynamic traffic load across the network, which suggests that handover decisions must be network-aware to prevent resource bottlenecks and ensure equitable service delivery [5].

Recent research on LEO handover management has taken two paths: machine learning models that require substantial computational resources and conventional heuristics that prioritize either signal quality or connection stability, but rarely both. Notably, few studies provide low-complexity hybrid strategies that balance these competing objectives while offering formal stability guarantees. Furthermore, existing approaches are typically validated under simplified orbital and channel assumptions rather than realistic operational conditions. In this paper, we propose the Hybrid Handover Strategy (HHS), a low-complexity algorithm that addresses these limitations by integrating multiple decision criteria with a hybrid trigger mechanism combining opportunistic and degradation-based switching.

While the HHS integrates multiple decision criteria, its formulation differs fundamentally from those of conventional multi-attribute decision making (MADM) approaches that rely on the static ranking of alternatives based on instantaneous attributes. In LEO handover control, decisions are temporally coupled. Switching too often leads to cumulative penalties. Furthermore, it makes connections less stable. The HHS explicitly takes this sequential nature into account by introducing a state-dependent hysteresis mechanism that incorporates the connection history into the decision process, rather than relying on per-epoch re-ranking. This approach enables stability-aware switching behavior. It also allows for the analytical characterization of ping-pong suppression. Such characterization is typically absent in classical MADM-based handover schemes.

This paper offers three main contributions:

1. We propose the HHS, a low-complexity algorithm that integrates a multi-attribute utility function with a hybrid trigger mechanism combining opportunistic and degradation-based switching. The algorithm incorporates a novel logistic-decay stability bonus to explicitly penalize unnecessary handovers and suppress the ping-pong effect.
2. We provide a formal mathematical analysis of the HHS, deriving distribution-free bounds on the handover trigger probability and a conservative two-event upper bound on the ping-pong probability. We also characterize the stability–quality trade-off and establish the algorithm’s computational and signaling complexity.
3. We validate the HHS using a high-fidelity Monte Carlo simulation driven by real-world two-line element (TLE) data from the operational Starlink constellation. Results demonstrate a 64% reduction in handover frequency compared to signal-to-interference-plus-noise ratio (SINR)-based benchmarks while maintaining 90.2% service availability.

The remainder of this paper is organized as follows. Section 2 reviews the related work. Section 3 presents the system model. Section 4 describes the proposed HHS algorithm. Section 5 provides the theoretical analysis. Section 6 presents the simulation results, and Section 7 concludes the paper.

## 2. Related Work

LEO handover management has changed from using single-metric triggers to using decision frameworks that have more than one goal. Recent studies have two main directions: low-complexity heuristic and multi-attribute models and computationally intensive artificial intelligence (AI) and machine learning (ML) techniques.

### 2.1. Advanced Heuristic and Multi-Attribute Strategies

Early handover strategies were based on intuitive metrics like the highest elevation or maximum visibility time. Recognizing the limitations of these single-metric approaches, recent research has focused on developing more nuanced MADM frameworks. A prominent approach is the use of utility theory, where a function maps multiple attributes to a single “desirability” score.

A significant illustration is the research conducted by Zhang et al., who utilize the Technique for Order Preference by Similarity to Ideal Solution (TOPSIS) method to equilibrate the communication delay and satellite load status [6]. Wang et al. also use utility theory to achieve feeder link handovers by creating a function that takes into account the backhaul capacity, service time, and a clear penalty for switching links too often [7]. While these methods offer a structured way to handle multiple criteria, their stability is crucial. Ngango et al.’s work includes a sensitivity analysis of different TOPSIS variants. It shows that a strong algorithm should not make random decisions based on noisy, real-time measurements [8]. The handover process could be modeled as a game that is spread out, instead of maximizing utility in a centralized way. Kim et al. put forward a learning-based auction system in which satellites bid to serve a user based on their own utility, which is determined by the signal strength and remaining service time [9]. This distributed architecture diminishes the signaling overhead inherent to centralized schemes. It thereby presents a viable alternative to the broadcast-based mechanisms frequently employed in practical models. Wang et al. extend utility-based optimization by proposing a conditional handover scheme [10]. This scheme jointly considers the service time and service capability through a reward function. To facilitate this, the authors construct a service continuity performance graph. This scheme allows them to predict and optimize handover sequences over the entire service duration [10].

### 2.2. Load-Aware Handover and Routing Mechanisms

Integrating the network load into handover decisions is now a well-established practice in the field. Modern research recognizes that, within constellations connected by intersatellite links (ISLs), the traffic load is not merely a local variable. Rather, it is a systemic characteristic that is deeply tied to the underlying routing architecture of the network. The term “load” is complex and has been represented in various forms, such as residual bandwidth on ISLs [11] and more comprehensive measures of resource utilization [12].

The most straightforward method to add load is to directly include it as a term in a utility function, which is demonstrated by Zhang et al. in their work [6]. A more sophisticated view, however, treats load balancing as a continuous, network-wide routing problem. The selective split load balancing (SSLB) strategy provided by Liu et al. exemplifies this, actively diverting traffic away from congested nodes [12]. This perspective suggests that handover and routing decisions are deeply coupled and should ideally be co-designed. A critical aspect of these approaches is how load information is disseminated. Centralized schemes require extensive signaling, whereas distributed mechanisms such as auction-based bidding or learning-based updates reduce the overhead but increase the algorithmic complexity. In contrast, many practical models assume the periodic broadcast of load information, which represents a lightweight compromise. Shinde et al. investigate distributed task allocation strategies for edge computing in LEO satellite Internet of Things (IoT) networks [13]. They demonstrate that these strategies reduce the processing latency and balance computational resources across satellites. Consequently, this work highlights the relevance of load-aware optimization beyond traditional handover decisions [13].

### 2.3. Intelligent Handover via AI and Machine Learning

Recent work applies AI, particularly deep reinforcement learning (DRL), to solve the complex, non-linear optimization problem of handover management. A notable trend is the shift to proactive, predictive control. The deep handover (DHO) protocol proposed by Lee et al. uses DRL to anticipate the need for a handover based on predictable orbital patterns, aiming to reduce the handover latency [14]. To manage the changing network topology, more advanced architectures use graph neural networks (GNNs) with DRL agents together to learn detailed representations of the current state of the network [15]. Recent studies have framed the issue as a multi-objective DRL task. These involve agents learning policies that jointly optimize the handover frequency, communication quality, and satellite load balancing. They frequently employ long short-term memory (LSTM) networks for traffic prediction [16]. Li et al. demonstrate a caching-aware DRL strategy that considers multiple attributes. This strategy includes the remaining service time and channel availability [17].

Fuzzy logic (FL) serves as a transparent alternative to the often opaque deep learning. By offering an interpretable, rule-based framework, fuzzy logic (FL) effectively manages uncertainty within the network. These principles are already well established in terrestrial 5G networks. In such environments, FL dynamically adjusts the handover parameters by evaluating the user velocity and signal quality. These proven methodologies are directly applicable to the challenges of the LEO context [18]. In contrast, while AI and ML models perform impressively in simulations, their real-world deployment faces significant hurdles. The primary limitations involve high computational complexity and a fundamental lack of interpretability [15,19]. For example, sophisticated frameworks like DRL combined with GNNs require immense training and processing resources. This makes them largely unsuitable for the resource-constrained hardware found on satellites. Furthermore, their “black box” nature makes them difficult to verify. In critical infrastructure, this lack of transparency is a major concern, as verifiability remains a paramount requirement.

### 2.4. Identifying the Research Gaps

A compelling tension exists within the current research, as detailed in Table 1. MADM and utility-based heuristics represent a practical approach focused on interpretability and efficiency. Conversely, AI/ML methods prioritize performance, aiming for near-optimal results at the cost of increased complexity. A third perspective involves distributed and auction-based methods. While these techniques contribute to better scalability, they also introduce significant challenges regarding signaling and coordination overhead. A further emerging direction is the shift from network-centric QoS metrics (e.g., SINR, availability) toward user-centric quality of experience (QoE) considerations, especially for streaming and multimedia applications, where short-term QoS degradations may not affect the perceived user experience. The integration of terrestrial networks (TNs) and NTNs in 6G systems introduces additional handover management complexities. One significant challenge is the limited effectiveness of reference signal received power (RSRP)-based triggers in large satellite coverage areas. Furthermore, overlapping terrestrial network (TN)-NTN regions often experience increased ping-pong effects [20].

In summary, MADM approaches are sensitive to parameter tuning [6], AI/ML solutions achieve high performance but require substantial resources [14,15,16], and distributed schemes introduce additional signaling overhead [9,11,12]. There is thus a need for solutions that are lightweight, interpretable, and validated under realistic orbital and channel models, while being inherently compatible with evolving QoE-oriented performance objectives. In terms of computational complexity, heuristic and MADM approaches typically scale linearly with the number of visible satellites, whereas AI/ML-based approaches incur orders of magnitude higher inference costs, underscoring the importance of maintaining low algorithmic overhead.

## 3. System Model

This section presents the system model for an LEO downlink. We consider a constellation described by real TLE data propagated with Simplified General Perturbations 4 (SGP4) to obtain satellite ephemerides and geometric visibility [21]. We specify the architecture and geometry, a comprehensive channel model with realistic impairments, and a spatiotemporal traffic/load model. These models enable the handover problem formulation evaluated in later sections.

### 3.1. System Architecture

Let $S=\{s_{1},\ldots ,s_{N}\}$ be the set of LEO satellites and let the ground terminal (GT) have geodetic coordinates (lat,lon). Denote by $r_{i}\left(t\right)\in R^{3}$ and $r_{\mathrm{GT}}\in R^{3}$ the Earth-centered, Earth-fixed coordinate (ECEF) positions of satellite $s_{i}$ and the GT, obtained from TLE with a standard SGP4 propagator [21]. The slant range is

(1) $$d_{i}\left(t\right)=∥r_{i}\left(t\right)-r_{\mathrm{GT}}∥.$$

**Figure 1:**
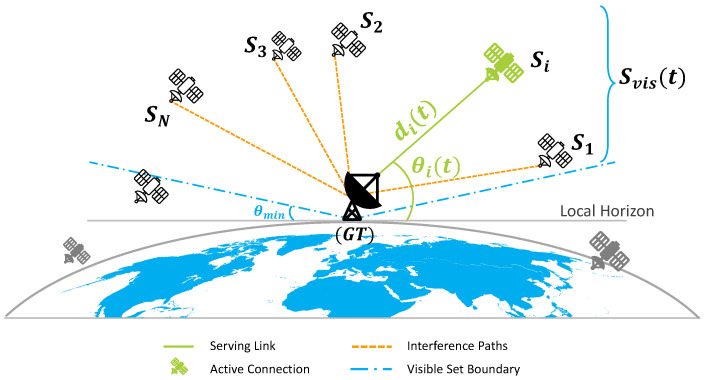
LEO satellite geometry with ground terminal (GT), visible satellites $S_{\mathrm{vis}}\left(t\right)$, elevation angle $\theta_{\min}$, and slant range $d_{i}\left(t\right)$.

Let $\hat{n}=r_{\mathrm{GT}}/∥r_{\mathrm{GT}}∥$ be the local zenith unit vector and ${\hat{e}}_{i}\left(t\right)=\left(r_{i}\left(t\right)-r_{\mathrm{GT}}\right)/d_{i}\left(t\right)$ the line-of-sight unit vector. The elevation angle is

(2) $$\theta_{i}\left(t\right)=\arcsin\;\left({\hat{e}}_{i}\left(t\right)\cdot \hat{n}\right),$$

and the visible set is $S_{\mathrm{vis}}\left(t\right)=\left\{s_{i}\in S:\theta_{i}\left(t\right)\geq \theta_{\min}\right\}$. Figure 1 illustrates this geometry, showing the ground terminal, visible satellites, elevation angle, and slant range.

### 3.2. Channel Model

The link quality experienced by the ground terminal is governed by a physically grounded link budget and the surrounding interference environment. We therefore describe the SINR, the thermal noise model, and the received-power/path loss decomposition, followed by the specific propagation impairments (free-space loss, atmospheric absorption, rain, shadowing/blockage, scintillation) and the co-channel interference model. Each component is combined to determine the instantaneous link quality.

We quantify the instantaneous link quality for a candidate serving satellite $s_{i}$ at time *t* by the SINR,

(3) $$\gamma_{i}\left(t\right)=\frac{P_{\mathrm{rx},i}\left(t\right)}{N_{0}+I_{\mathrm{total},i}\left(t\right)}.$$

where $P_{\mathrm{rx},i}\left(t\right)$ is the received signal power from $s_{i}$ at the GT, $N_{0}$ is the thermal noise power, and $I_{\mathrm{total},i}\left(t\right)$ is the aggregate co-channel interference at the GT. In this expression, all power quantities are in linear units (watts); the equivalent SINR in decibels is $\gamma_{i}\left(t\right)\;\left[\mathrm{dB}\right]=10\log_{10}\left(\gamma_{i}\left(t\right)\right)$. Throughout this section, link budget equations are presented in logarithmic form (dB/dBm) for convenience, with the understanding that linear conversions are applied when computing the SINR ratio above. For a receiver with bandwidth *B* and noise figure NF, the thermal noise power is given by

(4) $$\begin{array}{cc} N_{0}\;\left[W\right] & =k\;T_{0}\;B,\\ N_{0}\;\left[\mathrm{dBm}\right] & =-174+10\log_{10}B+\mathrm{NF}\;\left[\mathrm{dB}\right]. \end{array}$$

where *k* is Boltzmann’s constant and $T_{0}=290$ K is the reference noise temperature.

The received signal power follows a standard link budget relation and is given by

(5) $$P_{\mathrm{rx},i}\left(t\right)\;\left[\mathrm{dBm}\right]={\mathrm{EIRP}}_{i}\;\left[\mathrm{dBm}\right]+G_{t}\;\left[\mathrm{dBi}\right]-L_{\mathrm{total},i}\left(t\right)\;\left[\mathrm{dB}\right].$$

where ${\mathrm{EIRP}}_{i}$ denotes the satellite effective isotropic radiated power (EIRP) toward the user beam, $G_{t}$ is the GT antenna gain toward $s_{i}$, and $L_{\mathrm{total},i}\left(t\right)$ is the aggregate propagation loss in dB. We decompose this loss into physically distinct components:

(6) $$L_{\mathrm{total},i}\left(t\right)=L_{\mathrm{FSPL},i}\left(t\right)+A_{\mathrm{gas},i}\left(t\right)+A_{\mathrm{rain},i}\left(t\right)+L_{\mathrm{shadow},i}\left(t\right)-A_{\mathrm{scint},i}\left(t\right).$$

where $L_{\mathrm{FSPL},i}\left(t\right)$ is the free-space path loss at carrier frequency *f* and slant range $d_{i}\left(t\right)$; $A_{\mathrm{gas},i}\left(t\right)$ is atmospheric gaseous absorption computed per ITU-R P.676; $A_{\mathrm{rain},i}\left(t\right)$ is rain attenuation per ITU-R P.838 via the specific attenuation $\gamma_{R}\;=\;\kappa_{R}R^{\xi }$ and an elevation-dependent effective path length $L_{\mathrm{eff}}\left(\theta_{i},f\right)$; $L_{\mathrm{shadow},i}\left(t\right)$ models elevation-dependent urban/terrain shadowing or blockage consistent with 3GPP TR 38.811; and $A_{\mathrm{scint},i}\left(t\right)$ is tropospheric scintillation per ITU-R P.618 (implemented as a zero-mean random gain in dB, hence the minus sign). Equation (6) decomposes the total propagation loss into deterministic large-scale components and a stochastic scintillation term that captures short-term variability.

The free-space path loss (FSPL) depends only on the geometry and frequency:

(7) $$L_{\mathrm{FSPL},i}\left(t\right)\;\left[\mathrm{dB}\right]=20\log_{10}\left(\frac{4\pi f\;d_{i}\left(t\right)}{c}\right).$$

where *f* is the carrier frequency, $d_{i}\left(t\right)$ is the instantaneous slant range, and *c* is the speed of light.

Following ITU-R P.676, the specific gaseous attenuation (in dB/km) at frequency *f* (GHz) is

(8) $$\gamma_{\mathrm{gas}}\left(f\right)=0.1820\;f\left(N_{\mathrm{Oxygen}}^{\prime \prime }\left(f\right)+N_{\mathrm{Water}}^{\prime \prime }\left(f\right)\right),$$

where $N_{\mathrm{Oxygen}}^{\prime \prime }\left(f\right)$ and $N_{\mathrm{Water}}^{\prime \prime }\left(f\right)$ are the imaginary parts of the complex refractivities due to dry air (oxygen, plus small continuum terms) and water vapor, respectively. They are obtained from spectroscopic line summations:

(9) $$\begin{array}{cc} N_{\mathrm{Oxygen}}^{\prime \prime }\left(f\right) & =\sum_{i}S_{i}F_{i}\;+\;N_{D}^{\prime \prime }\left(f\right), \end{array}$$

(10) $$\begin{array}{cc} N_{\mathrm{Water}}^{\prime \prime }\left(f\right) & =\sum_{i}S_{i}F_{i}, \end{array}$$

where $S_{i}$ is the *i*th line strength, $F_{i}$ is the corresponding line-shape factor (including pressure/temperature broadening), and $N_{D}^{\prime \prime }\left(f\right)$ denotes the dry air continuum contribution (Debye spectrum of oxygen and pressure-induced nitrogen). Following ITU-R P.676 [22], the line strength is given by

(11) $$S_{i}=\left\{\begin{array}{cc} a_{1}\times 10^{-7}\;p_{d}\;\vartheta^{3}\exp\left[a_{2}\left(1-\vartheta \right)\right] & \mathrm{for}\;\mathrm{oxygen}\\ b_{1}\times 10^{-1}\;e_{w}\;\vartheta^{3.5}\exp\left[b_{2}\left(1-\vartheta \right)\right] & \mathrm{for}\;\mathrm{water}\;\mathrm{vapour} \end{array}\right.$$

where $p_{d}$ is the dry air pressure (hPa), $e_{w}$ is the water vapor partial pressure (hPa), ϑ=300/T is the inverse temperature ratio, *T* is the absolute temperature (K), and $\left(a_{1},a_{2}\right)$, $\left(b_{1},b_{2}\right)$ are spectroscopic coefficients tabulated in ITU-R P.676 Tables 1 and 2. The line-shape factor $F_{i}$ follows the Van Vleck–Weisskopf profile as detailed in ITU-R P.676 Annex 1. The slant-path gaseous attenuation in dB along the Earth–space path is then

(12) $$A_{\mathrm{gas},i}\left(t\right)=\int_{h_{1}}^{h_{2}}\frac{\gamma_{\mathrm{gas}}\left(f,h\right)}{\sin\phi (h)}\;dh\;\;\approx \;\;\sum_{\ell =1}^{L}a_{\ell }\;\gamma_{\mathrm{gas}}\left(f,h_{\ell }\right),$$

where ϕ(h) is the local apparent elevation angle along the path; the approximation partitions the atmosphere into exponentially increasing layers of thickness yielding path lengths $a_{\ell }$ through layer midpoints $h_{\ell }$ (layer-sum method recommended by ITU-R P.676 [22]). Rain attenuation is modeled using ITU-R P.838-3, where the specific attenuation (dB/km) follows a power-law function of rain rate *R* (mm/h):

(13) $$\gamma_{R}\;=\;\kappa_{R}\;R^{\xi }\;\left[\mathrm{dB}/\mathrm{km}\right],$$

where the coefficients $\kappa_{R}$ and ξ depend on the carrier frequency *f* (GHz), path elevation angle θ, and polarization tilt τ (relative to horizontal; $\tau =45^{{}^{\circ}}$ for circular). ITU-R P.838-3 provides frequency-dependent horizontal/vertical coefficients $\left(\kappa_{h},\xi_{h}\right)$ and $\left(\kappa_{v},\xi_{v}\right)$, which are combined for arbitrary path geometry/polarization as

(14) $$\kappa_{R}\;=\;\frac{1}{2}\;\left[\kappa_{h}+\kappa_{v}\;+\;\left(\kappa_{h}-\kappa_{v}\right)\;\cos^{2}\;\theta \;\cos\left(2\tau \right)\right],$$

(15) $$\xi =\frac{1}{2\kappa_{R}}\left[\kappa_{h}\xi_{h}+\kappa_{v}\xi_{v}+\left(\kappa_{h}\xi_{h}-\kappa_{v}\xi_{v}\right)\;\cos^{2}\;\theta \;\cos\left(2\tau \right)\right].$$

where $\left(\kappa_{h},\xi_{h}\right)$ and $\left(\kappa_{v},\xi_{v}\right)$ are obtained from ITU-R P.838’s frequency-fit formulae and tables [23]. The slant-path rain attenuation on the Earth–space link is then

(16) $$A_{\mathrm{rain},i}\left(t\right)\;=\;\gamma_{R}\left(f,R,\theta ,\tau \right)\;L_{\mathrm{eff}}\;\left(\theta_{i}\left(t\right),f\right),$$

where $L_{\mathrm{eff}}\left(\cdot \right)$ is the effective rain path length (km) for the link geometry (we adopt the standard Earth–space slant-path reduction used in propagation prediction methods). In (13)–(16), *R* is the local rain rate intersected by the instantaneous path to $s_{i}$, and $\theta =\theta_{i}\left(t\right)$ is the path elevation at time *t*.

In dense built environments, large-scale attenuation due to buildings is modeled following the 3GPP TR 38.811 urban shadowing framework. Specifically, the basic path loss includes a clutter term and a log-normal shadow-fading term:

(17) $$PL_{b}\left(d,f_{c},\alpha \right)=FSPL\left(d,f_{c}\right)+CL\left(\alpha ,f_{c}\right)+SF,\;SF∼N\left(0,\sigma_{SF}^{2}\right),$$

where $CL(\alpha ,f_{c})$ is the elevation- and frequency-dependent clutter loss, and SF is the large-scale shadow fading in dB. In LOS, $CL(\alpha ,f_{c})=0$ dB; in NLOS, both CL and $\sigma_{SF}$ are taken from the scenario/elevation tables in TR 38.811. Accordingly, the shadowing/blockage term used in our link budget is

(18) $$L_{\mathrm{shadow},i}\left(t\right)=CL_{i}\;\left(\theta_{i}\left(t\right),f_{c}\right)+SF_{i}\left(t\right),$$

where LOS/NLOS is drawn using the elevation-dependent LOS probabilities specified by TR 38.811, and $SF_{i}\left(t\right)∼N\left(0,\sigma_{SF}^{2}\right)$ captures the log-normal shadow-fading component for satellite $s_{i}$ at time *t* [24].

Tropospheric scintillation is modeled as per ITU-R P.618 [25] as a zero-mean Gaussian random variable with variance dependent on the frequency, elevation angle, and local climate:

(19) $$A_{\mathrm{scint},i}\left(t\right)∼N\;\left(0,\;\sigma_{i}^{2}\left(t\right)\right),$$

where $\sigma_{i}^{2}\left(t\right)$ is the time-varying variance. This formulation models scintillation as a zero-mean Gaussian process in dB, representing fast fluctuations in the received signal level that perturb the composite loss in Equation (6). The r.m.s. amplitude is

(20) $$\sigma_{i}\left(t\right)\;=\;\sigma_{\mathrm{ref}}\;f^{7/12}\;g\;\left(x\right)\;{\left[\sin\theta_{i}\left(t\right)\right]}^{-1.2}.$$

where *f* is the carrier frequency (GHz), $\theta_{i}\left(t\right)$ is the elevation angle, and $\sigma_{\mathrm{ref}}$ is a site-dependent reference r.m.s. from the local climate (per P.618). The aperture-averaging term uses $x=1.22\;D_{\mathrm{eff}}^{2}f^{2}/L$, with $D_{\mathrm{eff}}=\eta^{1/2}D$ being the effective antenna diameter, *L* the effective path length of the turbulent layer, and g(x) the P.618 aperture-averaging factor (all standard definitions in [25]).

Finally, co-channel interference aggregates contributions from non-serving satellites/beams that illuminate the ground terminal through main- or side-lobe coupling. Let $S_{\mathrm{co}}\left(t\right)$ be the set of co-channel, non-serving satellites. The total interference is

(21) $$I_{\mathrm{total},i}\left(t\right)=\sum_{s_{j}\in S_{\mathrm{co}}\left(t\right)}P_{\mathrm{rx},j\to \mathrm{GT}}\left(t\right),$$

where each $P_{\mathrm{rx},j\to \mathrm{GT}}\left(t\right)$ is computed via the same link budget formulation using the appropriate off-axis antenna patterns for both space and ground terminals. This completes the physical channel model that underpins the SINR in (3) and, in turn, the utility-based handover decisions evaluated in later sections.

In LEO satellite systems, the relative motion between satellites and ground terminals induces significant Doppler shifts. In this work, Doppler effects are assumed to be compensated for at the physical and link layers using standard frequency tracking and correction mechanisms. Such an approach is commonly adopted in contemporary LEO and NTN systems [26]. Consequently, the SINR values used by the proposed HHS algorithm represent post-compensation link quality estimates. Doppler therefore influences the handover process indirectly through its impact on the measured SINR dynamics, rather than being explicitly modeled at the decision layer.

### 3.3. Spatiotemporal Traffic Model

We represent the offered demand as a spatiotemporal field that couples the population density with the local time of day, yielding a heterogeneous, time-varying load across the satellites’ visible footprints. The Earth’s surface is partitioned into a grid G of cells indexed by *j*, each with area $A_{\mathrm{cell}}$ and center coordinates $\left({\mathrm{lat}}_{j},{\mathrm{lon}}_{j}\right)$. The baseline demand density is modeled as a latitude-dependent background augmented by urban “hotspots”:

(22) $$\rho_{\mathrm{pop}}\left(j\right)=\rho_{\mathrm{base}}\left({\mathrm{lat}}_{j}\right)+\sum_{m=1}^{M}\alpha_{m}\exp\;\left(-\frac{∥x_{j}-\mu_{m}{∥}_{2}^{2}}{2\sigma_{m}^{2}}\right),$$

where $x_{j}$ is the projected 2D location of cell *j*, $\mu_{m}$ are hotspot centers, $\sigma_{m}$ their spatial spreads, and $\alpha_{m}$ their amplitudes [2,27,28].

Temporal variation is captured by converting the simulation time to each cell’s local time and applying a simple diurnal multiplier. With $t_{\mathrm{UTC}}$ as the simulation time (hours),

(23) $$t_{\mathrm{local},j}=\left(t_{\mathrm{UTC}}+{\mathrm{lon}}_{j}/15\right)\;\mathrm{mod}\;24,$$

and the piecewise diurnal factor is

(24) $$M\left(t\right)=\left\{\begin{array}{cc} m_{\mathrm{night}}, & 0\leq t<6,\\ m_{\mathrm{day}}, & 6\leq t<17,\\ m_{\mathrm{eve}}, & 17\leq t<24, \end{array}\right.$$

where $0<m_{\mathrm{night}}\leq m_{\mathrm{day}}\leq m_{\mathrm{eve}}\leq 1$ set the off-peak, daytime, and evening activity levels, consistent with empirical usage patterns [29,30].

User activity within each cell drives demand. We let $U_{j}\left(t\right)∼\mathrm{Poisson}\;\left(\lambda_{j}\;M\left(t_{\mathrm{local},j}\right)\right)$, with 

(25) $$\lambda_{j}=\rho_{\mathrm{pop}}\left(j\right)\;A_{\mathrm{cell}}\;P_{\mathrm{pen}},$$

where $P_{\mathrm{pen}}\in \left[0,1\right]$ is the Internet penetration factor mapping the population to active users. For a per-user average bandwidth requirement $BW_{pp}$, the expected instantaneous demand contributed by cell *j* is

(26) $$\begin{array}{cc} D_{\mathrm{cell}}\left(j,t\right) & =E\left[U_{j}\left(t\right)\right]\;BW_{pp}\\ \; & =\rho_{\mathrm{pop}}\left(j\right)\;A_{\mathrm{cell}}\;P_{\mathrm{pen}}\;M\;\left(t_{\mathrm{local},j}\right)\;BW_{pp}, \end{array}$$

where $BW_{pp}$ (Mb/s per user) sets the per-user bandwidth demand.

The mapping from cell demand to satellite load uses the instantaneous visibility. Let $\theta_{i,j}\left(t\right)$ be the elevation of satellite $s_{i}$ as seen from cell *j*, and define the visibility indicator $V_{i}\left(j,t\right)=I\left\{\theta_{i,j}\left(t\right)\geq \theta_{\min}\right\}$. The demand incident on satellite $s_{i}$ is

(27) $$D_{i}\left(t\right)=\sum_{j\in G}V_{i}\left(j,t\right)\;D_{\mathrm{cell}}\left(j,t\right),$$

where $\theta_{\min}$ is the minimum elevation threshold (as defined in the geometry model).

Finally, the satellite load factor and its normalized complement used in utility terms are

(28) $$\rho_{i}\left(t\right)=\min\;\left(\frac{D_{i}\left(t\right)}{C_{\max}},\;1\right),\;{\tilde{\rho }}_{i}\left(t\right)=1-\rho_{i}\left(t\right),$$

where $C_{\max}$ denotes the satellite’s maximum data-handling capacity. This construction produces a dynamic and heterogeneous load field that aligns with geographic concentration and diurnal usage, stressing handover strategies under realistic spatiotemporal demands.

The satellite load indicator $\rho_{i}\left(t\right)$ is assumed to be available to the ground terminal through low-rate control-plane signaling. In practice, satellites can disseminate this information via periodic broadcast beacons or system information blocks. These transmissions convey coarse-grained load levels. Additionally, these values are averaged over suitable time windows. This assumption is consistent with existing load-aware access frameworks in satellite networks and does not require fine-grained or real-time scheduling states.

### 3.4. Problem Formulation

We model handover control as a sequential decision process over epochs t=0,Δt,2Δt,…,T. At each epoch, the controller selects one serving satellite from the visible set $S_{\mathrm{vis}}\left(t\right)$. Let $s*\left(t\right)\in S_{\mathrm{vis}}\left(t\right)$ denote the selected satellite. The cumulative handover count is

(29) $$N_{\mathrm{HO}}=\sum_{t>0}I\;\left\{\;s*\left(t\right)\neq s*\left(t-\Delta t\right)\right\},$$

where I{·} is the indicator function and Δt is the decision interval.

The goal is to balance instantaneous link quality with connection stability. We combine these two objectives in a single scalar criterion with non-negative policy weights $w_{q}$ and $w_{s}$:

(30) $$\underset{\left\{s*\left(t\right)\right\}}{\mathrm{maximize}}\;\sum_{t=0}^{T}w_{q}\;\gamma_{s*\left(t\right)}\left(t\right)\;-\;w_{s}\;N_{\mathrm{HO}}.$$

where $\gamma_{s*\left(t\right)}\left(t\right)$ is the serving-link SINR at time *t* as defined in the channel model. This formulation serves as a conceptual reference model expressing the fundamental trade-off between link quality and connection stability. The weights $w_{q}$ and $w_{s}$ are abstract scalarization parameters, whose ratio $w_{s}/w_{q}$ controls the relative penalty assigned to handovers versus SINR degradation. While the objective combines heterogeneous quantities originating from different physical domains (e.g., SINR in dB and handover counts), this is intentional at the conceptual level. In the practical algorithm implementation (Section 4), all metrics are mapped into a common dimensionless normalized scoring space prior to aggregation.

The decision must satisfy three feasibility constraints:

(31) $$\begin{array}{cccc} \gamma_{s*\left(t\right)}\left(t\right) & \geq \gamma_{\min}, & \; & \forall \;t\in [0,T], \end{array}$$

(32) $$\begin{array}{cccc} s*\left(t\right) & \in S_{\mathrm{vis}}\left(t\right), & \; & \forall \;t\in [0,T], \end{array}$$

(33) $$\begin{array}{cccc} \rho_{\;s*\left(t\right)}\left(t\right) & \leq \rho_{\max}, & \; & \forall \;t\in [0,T]. \end{array}$$

where $\gamma_{\min}$ is the minimum SINR requirement (a minimum quality-of-service threshold), $S_{\mathrm{vis}}\left(t\right)$ is the visibility set defined by the elevation constraint in the geometry model, $\rho_{\;s*\left(t\right)}\left(t\right)$ is the instantaneous load factor from the traffic model, and $\rho_{\max}<1$ reserves capacity headroom.

Solving the optimization problem in (30) is not tractable for a real-time system. The problem is non-causal. A globally optimal choice of s*(t) at the current step would require perfect knowledge of the entire future system state. This includes all future γ fluctuations, future load patterns, and future stochastic channel events. The search space is also combinatorial. It grows exponentially with the mission duration *T*. An exhaustive search is therefore infeasible. We use (30)–(33) as a reference model. We then evaluate a causal, low-complexity heuristic in Section 4.

## 4. Proposed Hybrid Handover Strategy (HHS)

The proposed algorithm seeks a principled balance between link quality and connection stability. Instead of applying hysteresis at the decision stage, the approach normalizes metrics into dimensionless scores and embeds continuity bias directly into the utility function. Each candidate satellite receives a multi-attribute score combining the normalized signal quality, geometry, and load. The incumbent’s score is boosted by a time-dependent stability bonus, while non-incumbents are penalized to discourage frequent switching. A new satellite must therefore overcome an explicit penalty before it can compete with the incumbent, and only then is a switching margin considered. The algorithm also retains standard cascade checks (forced service, opportunistic upgrade, reactive degradation). The algorithm’s contribution lies in embedding hysteresis directly into the utility scores.

### 4.1. Utility Formulation

We map heterogeneous metrics to [0,1] using monotone normalization with clipping, which improves the comparability and guards against outliers. For candidate $s_{i}$ at epoch *t*,

(34) $$q_{i}\left(t\right)=\min\;\left\{1,\max\;\left\{0,\frac{\gamma_{i}\left(t\right)-\gamma_{\min}}{\gamma_{\max}-\gamma_{\min}}\right\}\right\},$$

(35) $$e_{i}\left(t\right)=\min\;\left\{1,\max\;\left\{0,\frac{\theta_{i}\left(t\right)-\theta_{\min}}{\theta_{\max}-\theta_{\min}}\right\}\right\},$$

(36) $$\ell_{i}\left(t\right)={\tilde{\rho }}_{i}\left(t\right)=1-\rho_{i}\left(t\right)\in \left[0,1\right],$$

where $\gamma_{\min}$ and $\gamma_{\max}$ are fixed bounds used across experiments, $\theta_{\min}$ is the visibility cutoff from Section 3, $\theta_{\max}=90^{{}^{\circ}}$, and $\rho_{i}\left(t\right)$ denotes the per-satellite load with desirability ${\tilde{\rho }}_{i}\left(t\right)$ defined in Section 3. The base utility aggregates these components as

(37) $$U_{\mathrm{base},i}\left(t\right)=w_{\gamma }\;q_{i}\left(t\right)+w_{\theta }\;e_{i}\left(t\right)+w_{\mathrm{load}}\;\ell_{i}\left(t\right),$$

with non-negative weights $w_{\gamma },w_{\theta },w_{\mathrm{load}}$ that express the relative importance of the quality, geometry, and load. Since all inputs are clipped to [0,1], the base score is bounded and interpretable.

To promote stability within the scoring itself, we shape the utilities prior to selection. Let $i_{\mathrm{cur}}$ be the current serving satellite and $t_{\mathrm{conn}}$ the elapsed connection time. The stability bonus is modeled by a logistic function,

(38) $$\begin{array}{c} \psi \left(t_{\mathrm{conn}}\right)=\frac{1}{1+\exp\;\left(-\kappa \;[\;t_{\mathrm{conn}}-\tau \;]\right)}, \end{array}$$

where κ>0 and τ>0. The logistic function increases smoothly and saturates, rewarding persistence while preserving the possibility of switching. The final, shaped utility is

(39) $$U_{i}\left(t\right)=U_{\mathrm{base},i}\left(t\right)+I\left\{i=i_{\mathrm{cur}}\right\}\;w_{\mathrm{stab}}\;\psi \left(t_{\mathrm{conn}}\right)-I\left\{i\neq i_{\mathrm{cur}}\right\}\;P_{\mathrm{ho}},$$

where $w_{\mathrm{stab}}\geq 0$ scales the bonus and $P_{\mathrm{ho}}\geq 0$ is a fixed penalty applied to non-incumbents. Equation (39) produces a utility landscape that is explicitly biased toward continuity, so that only sufficiently advantageous alternatives can trigger a switch.

### 4.2. Decision Policy

At each decision epoch, the controller forms the visible set $S_{\mathrm{vis}}\left(t\right)=\left\{\;i:\theta_{i}\left(t\right)\geq \theta_{\min}\;\right\}$ and evaluates $U_{i}\left(t\right)$ for all $i\in S_{\mathrm{vis}}\left(t\right)$ using (39). The policy follows three rules in sequence. First, a forced-service reassignment is performed if the incumbent is no longer visible or its quality falls below $\gamma_{\min}$. Second, an opportunistic upgrade is allowed if a candidate exceeds the incumbent’s score by at least a margin $\Delta U_{\mathrm{th}}\geq 0$. This is to ensure that routine fluctuations do not trigger switching. Third, if the incumbent’s signal degrades below a threshold $\gamma_{\mathrm{th}}$, the best available candidate is selected. If none of these conditions hold, the incumbent is maintained. After a switch, the connection timer resets; otherwise, it advances by Δt. Ties in any maximization are broken by larger $U_{\mathrm{base},i}$, then larger $\gamma_{i}$, then larger $\theta_{i}$, and finally by the smallest index. The full procedure is summarized in Algorithm 1.
**Algorithm 1** Hybrid Handover Strategy (HHS)**Require:**   Set of Satellites: S; Epoch Duration: Δt   SINR Bounds: $\gamma_{\min}$, $\gamma_{\max}$; Elevation Bounds: $\theta_{\min}$, $\theta_{\max}$   Weights: $w_{\gamma }$, $w_{\theta }$, $w_{\mathrm{load}}$, $w_{\mathrm{stab}}$; Handover Penalty: $P_{\mathrm{ho}}$   Thresholds: $\Delta U_{\mathrm{th}}$, $\gamma_{\mathrm{th}}$; Load Factors: $\rho_{i}\left(t\right)$ for all i∈S   State: $\left(i_{\mathrm{cur}},t_{\mathrm{conn}}\right)$; Helper: clip(x,a,b)≜min{max{x,a},b}1:**for** each epoch *t* **do**2:      **Step 1: Visible Set**3:      $S_{\mathrm{vis}}\left(t\right)\leftarrow \left\{i\in S:\theta_{i}\left(t\right)\geq \theta_{\min}\right\}$4:      **Step 2: Compute Utilities**5:      **for** each $i\in S_{\mathrm{vis}}\left(t\right)$ **do**6:            $q_{i}\leftarrow \mathrm{clip}\left(\frac{\left(\gamma_{i}\left(t\right)-\gamma_{\min}\right)}{\left(\gamma_{\max}-\gamma_{\min}\right)},0,1\right)$7:            $e_{i}\leftarrow \mathrm{clip}\left(\frac{\left(\theta_{i}-\theta_{\min}\right)}{\left(\theta_{\max}-\theta_{\min}\right)},0,1\right)$8:            $\ell_{i}\leftarrow {\tilde{\rho }}_{i}$            ▹${\tilde{\rho }}_{i}=1-\rho_{i}\in \left[0,1\right]$9:            $U_{\mathrm{base},i}\leftarrow w_{\gamma }q_{i}+w_{\theta }e_{i}+w_{\mathrm{load}}\ell_{i}$10:          $U_{i}\leftarrow U_{\mathrm{base},i}+I\left[i=i_{\mathrm{cur}}\right]w_{\mathrm{stab}}\psi \left(t_{\mathrm{conn}}\right)-I\left[i\neq i_{\mathrm{cur}}\right]P_{\mathrm{ho}}$11:    **end for**12:    **Step 3: Selection Logic**13:    **if** $i_{\mathrm{cur}}\notin S_{\mathrm{vis}}\left(t\right)$ **or** $\gamma_{i_{\mathrm{cur}}}\left(t\right)<\gamma_{\min}$ **then**14:        $i*\leftarrow \arg\max_{i\in S_{\mathrm{vis}}\left(t\right)}U_{i}$        ▹ Forced service15:    **else if** $\exists \;i\in S_{\mathrm{vis}}\left(t\right):U_{i}-U_{i_{\mathrm{cur}}}\geq \Delta U_{\mathrm{th}}$ **then**16:        $i*\leftarrow \arg\max_{i:U_{i}-U_{i_{\mathrm{cur}}}\geq \Delta U_{\mathrm{th}}}U_{i}$    ▹ Opportunistic17:    **else if** $\gamma_{i_{\mathrm{cur}}}\left(t\right)<\gamma_{\mathrm{th}}$ **then**18:        $i*\leftarrow \arg\max_{i\in S_{\mathrm{vis}}\left(t\right)}U_{i}$   ▹ Reactive degradation19:    **else**20:        $i*\leftarrow i_{\mathrm{cur}}$             ▹ Maintain current21:    **end if**22:    **Step 4: Update State**23:    **if** $i*\neq i_{\mathrm{cur}}$ **then**24:        $i_{\mathrm{cur}}\leftarrow i*$;    $t_{\mathrm{conn}}\leftarrow 0$25:    **else**26:        $t_{\mathrm{conn}}\leftarrow t_{\mathrm{conn}}+\Delta t$27:    **end if**28:**end for**

## 5. Theoretical Analysis of the HHS Algorithm

This section analyzes the stability and efficiency of the proposed algorithm. We first establish distribution-free bounds on the handover trigger (margin exceedance) probability and then derive a conservative two-event upper bound on the ping-pong probability under an independence assumption. We also study the tightness of these bounds under Gaussian fluctuations. Next, we introduce an illustrative linearization that clarifies the trade-off induced by load weighting. Finally, we discuss the per-epoch computational complexity and place it in context with common alternatives.

### 5.1. Set-Up and Assumptions

We evaluate each decision epoch by comparing the shaped utility of the current serving satellite with the best available alternative. Let $U_{\mathrm{inc}}\left(t\right)$ be the shaped utility of the incumbent at time *t*, and let

(40) $$U_{\mathrm{alt}}\left(t\right)\;=\;\max_{\;i\neq i_{\mathrm{cur}}}\;U_{i}\left(t\right)$$

be the best competing option among the visible candidates. The decision signal is the utility gap

(41) $$\Delta U\left(t\right)\;≜\;U_{\mathrm{alt}}\left(t\right)-U_{\mathrm{inc}}\left(t\right),$$

where $i_{\mathrm{cur}}$ indexes the incumbent satellite, $U_{i}\left(t\right)$ is the shaped utility of candidate *i* at epoch *t*, and the maximization is taken over *i* in the visibility set $S_{\mathrm{vis}}\left(t\right)$ excluding the incumbent. The policy uses a positive margin $\Delta U_{\mathrm{th}}>0$ as an opportunistic switching threshold. A handover is considered only when ΔU(t) exceeds this margin.

Short-term variability in the gap is modeled as a random process over intervals with no handover activity. We write

(42) $$\mu \;=\;E\left\{\Delta U\left(t\right)\right\},\;\sigma^{2}\;=\;\mathrm{Var}\left\{\Delta U\left(t\right)\right\},$$

where μ and $\sigma^{2}$ denote the local mean and variance of ΔU(t). These are conditional moments defined over epochs where the connection remains stable. In this context, stability means that no handover occurs. We note that the connection time $t_{\mathrm{conn}}$ in (39) is formally a random stopping time, since handover decisions depend on stochastic utility fluctuations. By restricting attention to windows without switching, we obtain a well-defined local characterization of utility variability that is appropriate for analyzing near-term switching behavior. These statistics are estimated from data using the windowed procedure in (48).

### 5.2. Switching Probability Analysis: From Margin Exceedance to Ping-Pong

We analyze the probability of handover triggering in two stages using the conditional moments defined above. Since μ and $\sigma^{2}$ characterize utility variability over stable connection intervals, the resulting bounds apply within this conditional framework rather than providing unconditional global guarantees. First, we derive distribution-free bounds on single-epoch margin exceedance, which represents a necessary condition for any handover. Second, we extend this analysis to characterize the probability of ping-pong events, which require two consecutive handovers in opposite directions.

#### 5.2.1. Single-Epoch Margin Exceedance (Two-Sided Bound)

**Figure 2:**
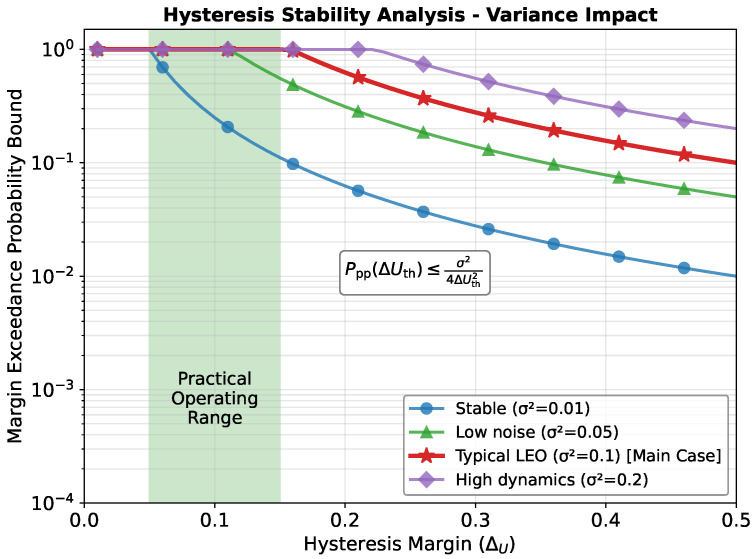
Single-epoch margin exceedance probability bound versus hysteresis margin ($\Delta_{U}$) under different utility variances. This bound represents a necessary condition for handover triggering.

Chebyshev’s inequality yields the distribution-free control of large deviations. For completeness, we first present a two-sided bound. A conservative sufficient condition for margin crossing is

(43) $$|\Delta U\left(t\right)-\mu |\;\geq \;2\;\Delta U_{\mathrm{th}},$$

where μ=E{ΔU(t)} is the local mean over windows without handovers. The resulting bound on the margin exceedance probability is

(44) $$\begin{array}{c} p_{\mathrm{exc}}\left(\Delta U_{\mathrm{th}}\right)\;\leq \;P\;\left(|\Delta U\left(t\right)-\mu |\geq 2\Delta U_{\mathrm{th}}\right)\;\leq \;\frac{\sigma^{2}}{\;4\Delta U_{\mathrm{th}}^{2}\;}. \end{array}$$

where $\sigma^{2}=\mathrm{Var}\left\{\Delta U\left(t\right)\right\}$ is the local variance. Since these are conditional moments estimated from stable intervals, this bound characterizes the exceedance probability within the conditional framework. In the common zero-mean approximation μ=0, (44) simplifies to $p_{\mathrm{exc}}\leq \sigma^{2}/\left(4\Delta U_{\mathrm{th}}^{2}\right)$, exhibiting the characteristic quadratic decay with the hysteresis margin, as illustrated in Figure 2.

When a non-zero bias is present, the same argument is combined with the reverse triangle inequality,

(45) $$P\;\left(|\Delta U(t)|\geq \Delta \right)\;\leq \;P\;\left(|\Delta U(t)-\mu |\geq \Delta -|\mu |\right)\;\left(\Delta >|\mu |\right).$$

Substituting $\Delta =2\Delta U_{\mathrm{th}}$ gives a bias-aware bound,

(46) $$p_{\mathrm{exc}}\left(\Delta U_{\mathrm{th}}\right)\;\leq \;\frac{\sigma^{2}}{{\left(2\Delta U_{\mathrm{th}}-\left|\mu \right|\right)}^{2}}\;\mathrm{for}\;2\Delta U_{\mathrm{th}}>\left|\mu \right|.$$

Thus, this shows a graceful degradation as |μ| increases. The statistics μ and $\sigma^{2}$ are estimated from a sliding window of epochs with no switch. The estimators are

(47) $$\bar{\Delta U}=\frac{1}{W}\sum_{w=1}^{W}\Delta U\left(t_{w}\right),$$

(48) $${\hat{\sigma }}^{2}=\frac{1}{W-1}\sum_{w=1}^{W}{\left(\Delta U\left(t_{w}\right)-\bar{\Delta U}\right)}^{2},$$

where ${\left\{t_{w}\right\}}_{w=1}^{W}$ is the window of decision times. A robust alternative based on the median absolute deviations may be used if outliers are present.

#### 5.2.2. One-Sided Bound (Cantelli’s Inequality)

The two-sided Chebyshev bound in (44) is conservative for the HHS algorithm because the opportunistic trigger in Algorithm 1 activates only when $\Delta U\left(t\right)\geq \Delta U_{\mathrm{th}}$, i.e., a one-sided exceedance. The negative deviation $\Delta U\left(t\right)\leq -\Delta U_{\mathrm{th}}$ (incumbent significantly better than alternatives) does not trigger any handover and is therefore irrelevant to switching behavior.

A tighter analysis uses the one-sided Cantelli inequality. For a random variable *X* with mean μ and variance $\sigma^{2}$, Cantelli’s inequality states

(49) $$P\left(X-\mu \geq k\sigma \right)\;\leq \;\frac{1}{1+k^{2}},\;k>0.$$

Applying this to the utility gap ΔU(t) with $k=(\Delta U_{\mathrm{th}}-\mu )/\sigma$ yields

(50) $$p_{\mathrm{trigger}}\left(\Delta U_{\mathrm{th}}\right)\;≜\;P\left(\Delta U\left(t\right)\geq \Delta U_{\mathrm{th}}\right)\;\leq \;\frac{\sigma^{2}}{\sigma^{2}+{\left(\Delta U_{\mathrm{th}}-\mu \right)}^{2}}.$$

For the zero-mean case (μ=0), this simplifies to

(51) $$p_{\mathrm{trigger}}\left(\Delta U_{\mathrm{th}}\right)\;\leq \;\frac{\sigma^{2}}{\sigma^{2}+\Delta U_{\mathrm{th}}^{2}}.$$

This bound is tighter than the two-sided Chebyshev bound in (44) and directly corresponds to the algorithm’s one-sided triggering mechanism.

#### 5.2.3. Two-Event Ping-Pong Probability

A ping-pong event occurs when the terminal switches from satellite *A* to satellite *B* and subsequently switches back to *A* within a short time window. For analytical tractability, we consider a two-satellite local competition model where *B* represents the dominant alternative at the time of switching; this approximation captures the essential two-crossing dynamics while remaining consistent with the general multi-candidate formulation. This requires two conditions to be satisfied.

1. At epoch *t*: The utility gap $\Delta U_{A\to B}\left(t\right)=U_{B}\left(t\right)-U_{A}\left(t\right)\geq \Delta U_{\mathrm{th}}$, triggering a switch to *B*.
2. At epoch $t^{\prime }>t$: After the roles swap (B is now incumbent), the reverse gap $\Delta U_{B\to A}\left(t^{\prime }\right)=U_{A}\left(t^{\prime }\right)-U_{B}\left(t^{\prime }\right)\geq \Delta U_{\mathrm{th}}$, triggering a switch back to *A*.

Let $X\left(t\right)=V_{B}\left(t\right)-V_{A}\left(t\right)$ denote the base utility gap before stability shaping. The first switch requires X(t) to exceed a positive threshold (B appears better), while the second switch requires $X(t^{\prime })$ to fall below a negative threshold (A appears better). Thus, ping-pong requires the base utility gap to cross thresholds in both directions.

Under the conservative assumption of independence between decision epochs, the ping-pong probability is bounded by

(52) $$p_{\mathrm{pp}}\left(\Delta U_{\mathrm{th}}\right)\;\leq \;p_{\mathrm{trigger}}^{\left(1\right)}\times p_{\mathrm{trigger}}^{\left(2\right)},$$

where $p_{\mathrm{trigger}}^{\left(1\right)}$ and $p_{\mathrm{trigger}}^{\left(2\right)}$ are the probabilities of the first and second threshold crossings, respectively. Applying the Cantelli bound (51) to each event,

(53) $$p_{\mathrm{pp}}\left(\Delta U_{\mathrm{th}}\right)\;\leq \;{\left(\frac{\sigma^{2}}{\sigma^{2}+\Delta U_{\mathrm{th}}^{2}}\right)}^{2}.$$

This bound exhibits $O(1/\Delta U_{\mathrm{th}}^{4})$ decay for large margins, which is significantly faster than the $O(1/\Delta U_{\mathrm{th}}^{2})$ decay of single-event bounds. The independence assumption is conservative: in practice, a positive temporal correlation in the utility gap would further reduce the ping-pong probability, making (53) an upper bound. This is because ping-pong requires a sign reversal in the utility gap, and a positive correlation reduces the likelihood of such reversals over short time horizons.

We emphasize that the single-epoch bounds in (44) and (50) represent necessary conditions for ping-pong. If the probability of a single margin crossing is low, ping-pong events are necessarily rare. The two-event bound in (53) provides more direct characterization of the ping-pong probability by explicitly modeling the two-crossing requirement.

### 5.3. Gaussian Tightness and Asymptotics

The ping–pong event is now studied under a Gaussian fluctuation model for the utility gap. Assume $\Delta U\left(t\right)∼N\left(\mu ,\sigma^{2}\right)$, and consider the two-sided exceedance that triggers a margin crossing within two epochs. The exact probability is

(54) $$\begin{array}{c} p_{\mathrm{pp}}^{N}\left(\Delta U_{\mathrm{th}}\right)=\Phi \;\left(\frac{-2\Delta U_{\mathrm{th}}-\mu }{\sigma }\right)+1-\Phi \;\left(\frac{2\Delta U_{\mathrm{th}}-\mu }{\sigma }\right), \end{array}$$

where Φ(·) is the standard normal cumulative distribution function, μ=E{ΔU(t)}, and $\sigma^{2}=\mathrm{Var}\left\{\Delta U\left(t\right)\right\}$. When μ=0, (54) reduces to

(55) $$p_{\mathrm{pp}}^{N}\left(\Delta U_{\mathrm{th}}\right)=2\;Q\;\left(\frac{2\Delta U_{\mathrm{th}}}{\sigma }\right),$$

where Q(x)=1−Φ(x) is the Gaussian tail function.

**Figure 3:**
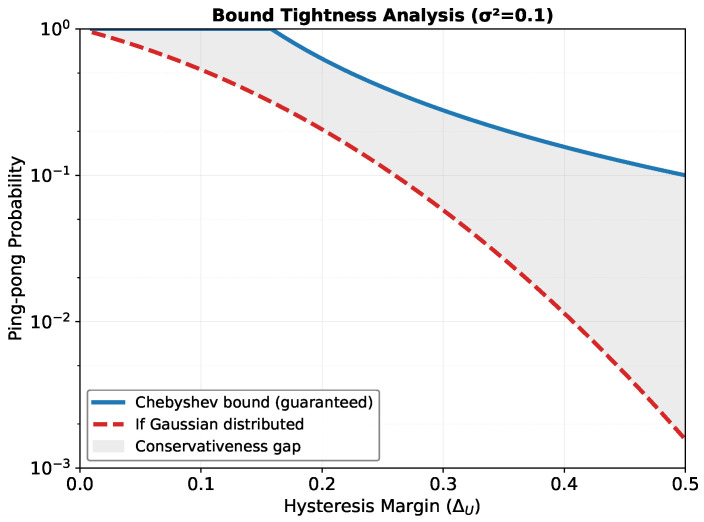
Comparison of Chebyshev and Gaussian bounds for $\sigma^{2}=0.1$. The shaded region indicates the worst-case safety margin provided by the distribution-free Chebyshev bound relative to the tighter Gaussian-specific bound.

For large arguments, the tail admits the standard asymptotic form $Q\left(x\right)\approx \frac{1}{x\sqrt{2\pi }}\;e^{-x^{2}/2}$. Substituting $x=2\Delta U_{\mathrm{th}}/\sigma$ gives

(56) $$p_{\mathrm{pp}}^{N}\left(\Delta U_{\mathrm{th}}\right)\;\approx \;\frac{1}{\sqrt{2\pi }}\;\frac{\sigma }{\Delta U_{\mathrm{th}}}\;\exp\;\left(-\frac{2\Delta U_{\mathrm{th}}^{2}}{\sigma^{2}}\right),$$

which makes the exponential suppression explicit when the margin dominates the standard deviation. Figure 3 compares this Gaussian-specific prediction with the distribution-free Chebyshev bound. It illustrates the gap between generality and tightness.

### 5.4. Illustrative Load–Quality Linearization

Load-aware scoring discourages association with congested satellites, which reduces the switching pressure, but it can move the selection away from the strongest link. To isolate this trade-off, we consider the load weight $w_{\mathrm{load}}$ and define two performance summaries: the expected handover rate $\Gamma (w_{\mathrm{load}})$ and an average link quality metric $\bar{Q}\left(w_{\mathrm{load}}\right)$ (for example, mean γ over epochs). A first-order Taylor expansion about a reference $w_{0}$ gives

(57) $$\begin{array}{cc} \Gamma \;\left(w_{\mathrm{load}}\right) & \approx \Gamma_{0}-k\;\left(w_{\mathrm{load}}-w_{0}\right), \end{array}$$

(58) $$\begin{array}{cc} \bar{Q}\;\left(w_{\mathrm{load}}\right) & \approx {\bar{Q}}_{0}-\beta \;\left(w_{\mathrm{load}}-w_{0}\right), \end{array}$$

where $\Gamma_{0}=\Gamma \left(w_{0}\right)$ and ${\bar{Q}}_{0}=\bar{Q}\left(w_{0}\right)$ are the baseline values at the reference point.

The local slopes quantify the immediate exchange between stability and link quality:

(59) $$k≜-{\left.\frac{d\Gamma }{dw_{\mathrm{load}}}\right|}_{w_{0}}\geq 0,\;\beta ≜{\left.\frac{d\;\bar{Q}}{dw_{\mathrm{load}}}\right|}_{w_{0}}\geq 0,$$

where *k* measures the reduction in handover rate per unit increase in $w_{\mathrm{load}}$ and β measures the reduction in average link quality per unit increase in $w_{\mathrm{load}}$.

**Figure 4:**
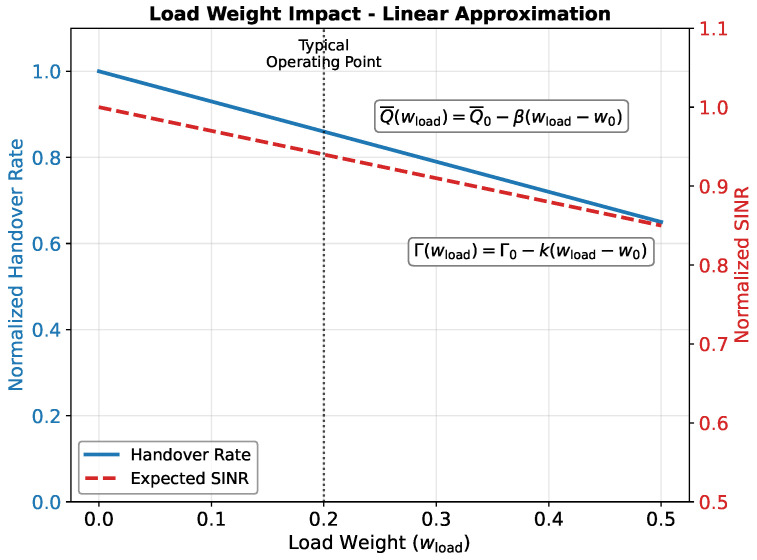
Impact of load weight ($w_{\mathrm{load}}$) on handover rate and SINR. Increasing the load weight reduces the handover frequency by prioritizing less congested satellites.

Figure 4 illustrates the qualitative behavior of (57)–(59). These linearizations approximate the trade-off trends observed in simulations but do not predict absolute values. In experiments, *k* and β are estimated by finite differences on a validation slice using a small step δ>0:

(60) $$k\;\approx \;\frac{\Gamma \left(w_{0}\right)-\Gamma \left(w_{0}+\delta \right)}{\delta },$$

(61) $$\beta \;\approx \;\frac{\bar{Q}\left(w_{0}+\delta \right)-\bar{Q}\left(w_{0}\right)}{\delta }.$$

### 5.5. Sensitivity to Stability Reward Parameters

The preceding analysis established distribution-free bounds on margin exceedance and the ping-pong probability as functions of the threshold $\Delta U_{\mathrm{th}}$ and utility variance $\sigma^{2}$. We now extend this framework to quantify the stability reward parameters. Specifically, we examine how the stability weight $w_{\mathrm{stab}}$, steepness κ, and midpoint τ of the logistic bonus influence switching behavior.

Recall from (39) that the shaped utilities are

(62) $$U_{\mathrm{alt}}=U_{\mathrm{base},\mathrm{alt}}-P_{\mathrm{ho}},\;U_{\mathrm{inc}}=U_{\mathrm{base},\mathrm{inc}}+w_{\mathrm{stab}}\;\psi \left(t_{\mathrm{conn}}\right),$$

where $\psi \left(t_{\mathrm{conn}}\right)=1/\left(1+e^{-\kappa (t_{\mathrm{conn}}-\tau )}\right)$ is the logistic stability bonus. The utility gap can therefore be written as

(63) $$\Delta U\left(t\right)=\Delta U_{\mathrm{base}}\left(t\right)-P_{\mathrm{ho}}-w_{\mathrm{stab}}\;\psi \left(t_{\mathrm{conn}}\right),$$

where $\Delta U_{\mathrm{base}}\left(t\right)≜U_{\mathrm{base},\mathrm{alt}}-U_{\mathrm{base},\mathrm{inc}}$ is the base utility gap before stability shaping. An opportunistic handover is triggered when $\Delta U\left(t\right)\geq \Delta U_{\mathrm{th}}$, which can be rearranged as a threshold condition on the base gap:

(64) $$\Delta U_{\mathrm{base}}\left(t\right)\;\geq \;\underset{c(t_{\mathrm{conn}})}{\underset{︸}{\Delta U_{\mathrm{th}}+P_{\mathrm{ho}}+w_{\mathrm{stab}}\;\psi \left(t_{\mathrm{conn}}\right)}}.$$

The effective threshold $c(t_{\mathrm{conn}})$ increases with the stability weight $w_{\mathrm{stab}}$ and with the connection time $t_{\mathrm{conn}}$ (through ψ), making switching progressively more difficult as the connection matures.

Applying the one-sided Cantelli bound from Section 5.2 to the base gap $\Delta U_{\mathrm{base}}\left(t\right)$, which has mean μ and variance $\sigma^{2}$, yields

(65) $$p_{\mathrm{trigger}}\left(t_{\mathrm{conn}}\right)\;\leq \;\frac{\sigma^{2}}{\sigma^{2}+{\left(c(t_{\mathrm{conn}})-\mu \right)}^{2}},$$

where $c\left(t_{\mathrm{conn}}\right)=\Delta U_{\mathrm{th}}+P_{\mathrm{ho}}+w_{\mathrm{stab}}\;\psi \left(t_{\mathrm{conn}}\right)$. This bound directly quantifies how $w_{\mathrm{stab}}$, κ, and τ reduce the trigger probability: a larger $w_{\mathrm{stab}}$ increases *c*, while a larger κ accelerates the saturation of ψ, and a smaller τ shifts the bonus buildup to earlier connection times.

**Figure 5:**
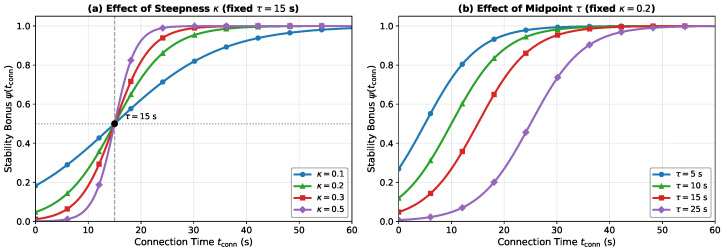
Logistic stability bonus function $\psi (t_{\mathrm{conn}})$. (**a**) Effect of steepness parameter κ with fixed midpoint τ=15 s. (**b**) Effect of midpoint parameter τ with fixed steepness κ=0.2.

Figure 5 illustrates the logistic stability bonus $\psi (t_{\mathrm{conn}})$ for different values of κ and τ. Higher steepness κ produces a sharper transition from a low to high bonus, while a larger τ delays the onset of the stability reward. The baseline parameters (κ=0.2, τ=15 s) provide a gradual buildup that reaches near-saturation within approximately 40 s of continuous connection.

**Figure 6:**
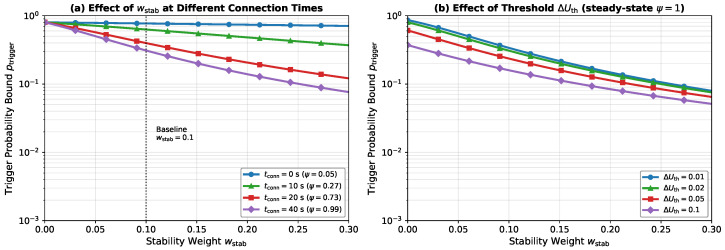
Sensitivity of the trigger probability bound to stability reward parameters. (**a**) Effect of $w_{\mathrm{stab}}$ at different connection times $t_{\mathrm{conn}}$, showing how the accumulated stability bonus reduces the switching likelihood. (**b**) Effect of the opportunistic threshold $\Delta U_{\mathrm{th}}$ at steady state (ψ=1). Parameters: $\sigma^{2}=0.01$, μ=0, $P_{\mathrm{ho}}=0.03$, κ=0.2, τ=15 s.

Figure 6 shows the sensitivity of the trigger probability bound (65) to the stability weight $w_{\mathrm{stab}}$. In Figure 6a, the curves correspond to different connection times, demonstrating that longer connections (higher ψ) yield substantially lower trigger probabilities. At the baseline operating point ($w_{\mathrm{stab}}=0.1$, $t_{\mathrm{conn}}=40$ s), the bound drops below $10^{-1}$. This result demonstrates that opportunistic switching is strongly suppressed. Figure 6b shows the interaction with the threshold $\Delta U_{\mathrm{th}}$. Higher thresholds provide an additional margin. This margin compounds with the stability reward. Consequently, the likelihood of switching decreases further.

Handovers occur only when the trigger condition is satisfied. Therefore, the bound on $p_{\mathrm{trigger}}$ serves as an analytical upper limit on the per-epoch switching probability. This relationship characterizes the sensitivity of the switching behavior to the stability reward parameters. This analysis confirms the effectiveness of the logistic bonus mechanism. By using appropriate choices for $w_{\mathrm{stab}}$, κ, and τ, we achieve effective and quantifiable control over the handover frequency.

### 5.6. Per-Epoch Complexity and Context

Let $N_{v}=\left|S_{\mathrm{vis}}\left(t\right)\right|$ be the number of visible satellites at an epoch. Computing the normalized components $\left(q_{i},e_{i},\ell_{i}\right)$ and the shaped utilities $U_{i}$ is constant work per candidate, so the utility pass is $O(N_{v})$. Selecting the best candidate is an argmax over at most $N_{v}$ values, which is also $O(N_{v})$. Hence, the overall per-epoch complexity of the HHS is $O(N_{v})$ with small constants.

For context, classical MADM ranking such as TOPSIS requires normalization across *M* metrics and distance computations to ideal points, which is $O(MN_{v})$ with an optional ranking cost $O(N_{v}\log N_{v})$. In contrast, learning-based methods (for example, deep reinforcement learning) incur the cost of network inference per epoch, which can be characterized by the total multiply–add count of the model and its memory footprint; this is hardware-dependent and typically of a higher cost than the arithmetic used in the HHS. Finally, the signaling overhead for load awareness can be realized via periodic broadcast beacons, adding only small per-epoch overhead proportional to $N_{v}$.

The HHS operates at the decision layer. It determines when a handover should occur and which satellite to select. However, the actual handover execution latency depends on the underlying access protocol. Therefore, this latency falls outside the algorithmic scope. The algorithm requires three input categories: (i) SINR estimates, obtainable from standard downlink measurements; (ii) elevation angles, computable from publicly available ephemeris data; and (iii) per-satellite load factors, disseminated via periodic broadcast beacons as assumed in the traffic model. The HHS requires no closed-loop signaling or additional measurement reports beyond standard link quality feedback. This design ensures compatibility with existing satellite access frameworks. Furthermore, it imposes no additional signaling burden on the system.

## 6. Performance Evaluation

This section presents a comprehensive evaluation of the proposed HHS against the benchmark strategies, which is achieved through an extensive Monte Carlo simulation campaign. The simulation framework, which is explained in more detail in the next subsections, is designed to be a high-fidelity representation of a modern LEO network. It uses the complete channel and spatiotemporal traffic models from Section 3. The goal of this evaluation is to carefully look at and measure how well each algorithm works on a set of important metrics. This will give a clear and data-driven picture of the trade-offs between service reliability, link quality, and connection stability.

### 6.1. Simulation Set-Up

The performance of the handover algorithms is assessed through a discrete-time Monte Carlo simulation. Every run within this environment represents a continuous LEO downlink session. The simulation utilizes the geometry, channel, and traffic models detailed in Section 3 to ensure accuracy. A summary of all configuration settings and algorithm parameters is available in Table 2.

#### 6.1.1. Simulation Framework and Methodology

The evaluation utilizes a custom-built, discrete-time simulation framework developed in Python (version 3.12). Performance is measured across 50 Monte Carlo runs for each algorithm, resulting in a total of 200 simulations. Each run represents two hours of continuous network operation, modeled with a 10 s time-step resolution. To ensure that the results are not biased by a single orbital configuration, the start time for each run is randomized within a seven-day window. This approach introduces essential temporal diversity into the initial satellite positions. Furthermore, all stochastic processes are seeded independently. These processes include shadowing, scintillation, rain, and traffic patterns. Such independent seeding is necessary to guarantee statistical independence across all simulation runs.

#### 6.1.2. System Architecture and Configuration

The theoretical models defined in Section 3 are translated into a simulation environment using realistic parameters. These settings ensure that the model accurately represents a modern LEO network deployment. All necessary configuration and algorithm parameters are organized in Table 2.

- The framework uses the Starlink constellation, with orbital parameters derived from a real-time TLE dataset provided by CelesTrak [31]. To ensure that only operational satellites are considered, the dataset is filtered to a set of satellites that meet strict criteria: mean motion between 14.9 and 15.2 revolutions per day (corresponding to an approximate 550 km altitude), a low drag coefficient (|β*|<0.0001), and a TLE age of less than 30 days. Satellite positions are computed using the standard SGP4 propagator via the Skyfield (version 1.53) library [32].
- The simulation models a stationary GT in Riyadh, Saudi Arabia. Link budget and traffic parameters follow the models in Section 3. All key system parameters for the link budget and network capacity are summarized in Table 2.

The HHS parameters in Table 2 are selected based on system-level considerations and validated through sensitivity analysis. The quality weights are defined as $w_{\gamma }=0.5$, $w_{\theta }=0.15$, and $w_{\mathrm{load}}=0.25$. These values reflect the relative importance of the instantaneous link quality, satellite geometry, and load balancing. Together, they function to maintain service continuity. Notably, this configuration assigns the dominant role to the link quality. The stability weight $w_{\mathrm{stab}}=0.1$ and handover penalty $P_{\mathrm{ho}}=0.03$ are chosen to introduce a moderate persistence bias toward the incumbent link without preventing timely transitions when a sustained quality advantage exists. The opportunistic margin is set to $\Delta U_{\mathrm{th}}=0.02$. This value corresponds to a small relative utility difference. It effectively suppresses handovers driven by short-term fluctuations. Simultaneously, it preserves responsiveness to meaningful improvements. The logistic stability parameters are set to κ=0.2 and τ=15 s. These values yield a gradual stability bonus. The bonus increases during the early connection phase and saturates thereafter. This pattern reflects typical LEO handover dynamics. The robustness of these parameter choices and their impacts on switching behavior are quantitatively examined in Section 5.4.

#### 6.1.3. Benchmark Handover Strategies

We compare the HHS against three common strategies: Longest Visibility Time (LVT), the Highest Elevation Angle (HEA), and the Highest SINR algorithm. The LVT strategy represents a highly conservative, stability-focused approach, designed to be resistant to transient fluctuations. LVT uses a time-to-trigger (TTT) of 240ms. Since 0.24s≪Δt, we emulate TTT with a one-epoch persistence check together with a hysteresis margin (set to 3.0 dB) so that a candidate must remain superior for at least one full epoch before switching [18]. The HEA strategy represents a classic, low-complexity, geometry-based approach. HEA selects the highest-elevation visible satellite at each epoch. A handover is triggered if a candidate satellite’s elevation angle, $\theta_{j}$, exceeds that of the current satellite, $\theta_{i}$, by a hysteresis margin of 4.0° [33]. The Highest SINR strategy serves as our modern greedy baseline, designed to aggressively maximize the instantaneous link quality. The Highest SINR algorithm greedily selects the candidate with the largest instantaneous SINR. It triggers a handover if a candidate satellite’s SINR exceeds the current link by a 2.0 dB hysteresis margin [2].

### 6.2. Results and Discussion

#### 6.2.1. Comparative Performance Analysis

The comprehensive performance metrics for each handover algorithm are summarized in Table 3. These results are aggregated from 50 Monte Carlo simulations. The proposed HHS algorithm achieves a mean of 140.9 handovers. This performance represents a 64.0% reduction compared to the Highest SINR algorithm, which records 390.9 handovers. Similarly, the HHS shows a 54.4% reduction relative to the HEA benchmark of 309.2 handovers. Notably, this substantial decrease in handover frequency is achieved without sacrificing link quality. The algorithm maintains a mean SINR of 8.8 dB and service availability of 90.2%.

**Figure 7:**
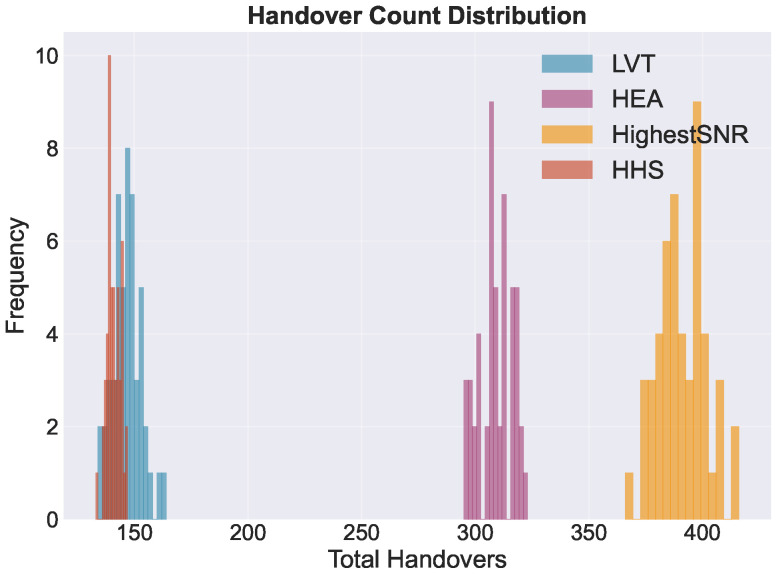
Distribution of total handover counts across algorithms over all Monte Carlo runs. Histograms illustrate the variability in switching behavior, with HHS and LVT concentrated in the low-handover regime (approximately 140–150 handovers), while HEA and Highest SINR exhibit substantially higher switching frequencies.

Figure 7 displays the distribution of handover counts across all Monte Carlo runs. This visualization reveals distinct operational regimes among the evaluated strategies. Stability-focused algorithms, specifically the HHS and LVT, tend to cluster within the 140 to 150 handover range. In contrast, quality-focused strategies such as the HEA and Highest SINR algorithm exhibit much higher switching rates. These strategies typically fall within the 300 to 400 range. Among the stability-oriented approaches, the proposed HHS demonstrates the highest level of consistency. It maintains the lowest variance, as evidenced by a standard deviation of ±3.1. This narrow distribution indicates that the performance of the HHS remains predictable across varying orbital configurations. We observe low variance combined with a hysteresis margin of $\Delta U_{\mathrm{th}}=0.02$. This leads to a lower handover trigger probability, as detailed in Section 5. Therefore, this approach effectively suppresses spurious switching events.

**Figure 8:**
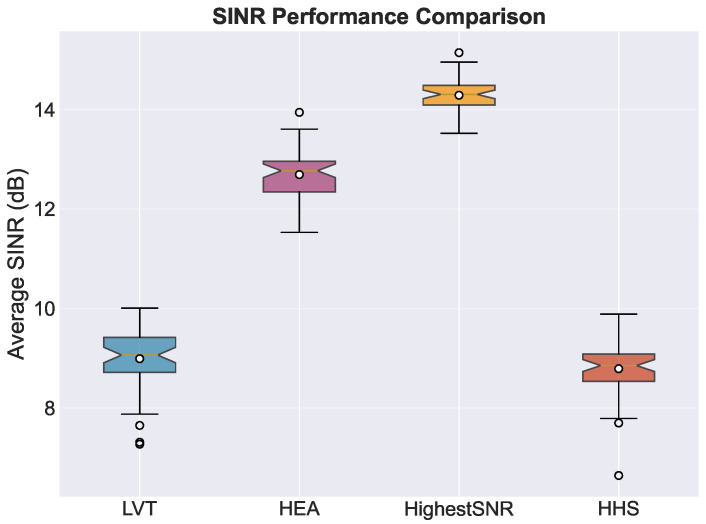
Average SINRs of the connected satellite across algorithms. Highest SINR achieves the best link quality by design, while stability-focused strategies (HHS, LVT) trade some SINR performance for reduced handover frequencies.

Figure 8 shows the SINR performance. It shows that the HHS has a median SINR of about 8.8 dB and an interquartile range of 8.2 to 9.4 dB. This is a 5.5 dB drop from the highest SINR (14.3 dB), but the SINR levels that are reached are still well above the normal operational limits for LEO satellite communications. The notched boxplot shows that the median SINR values between algorithms are statistically different from each other. This confirms the trade-off between link quality and connection stability.

**Figure 9:**
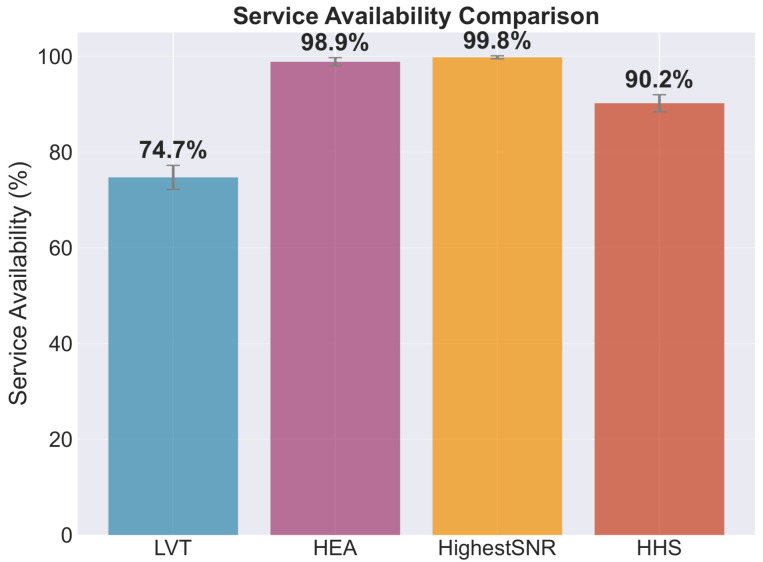
Service availability across algorithms, defined as the fraction of time for which the connected link exceeds the minimum SINR threshold $\gamma_{\min}$. HHS maintains high availability (90.2%) while significantly reducing the handover frequency compared to stability-only strategies.

Service availability emerges as the critical differentiator among stability-focused strategies. As shown in Figure 9, while LVT achieves a comparable handover rate (146.4), its service availability of 74.7% falls significantly below operational requirements. In contrast, the HHS maintains 90.2% availability, demonstrating that the multi-criteria utility function successfully balances stability with service continuity. The quality-focused algorithms achieve near-perfect availability (98.9% for HEA, 99.8% for Highest SINR), but at the cost of an excessive handover frequency.

**Figure 10:**
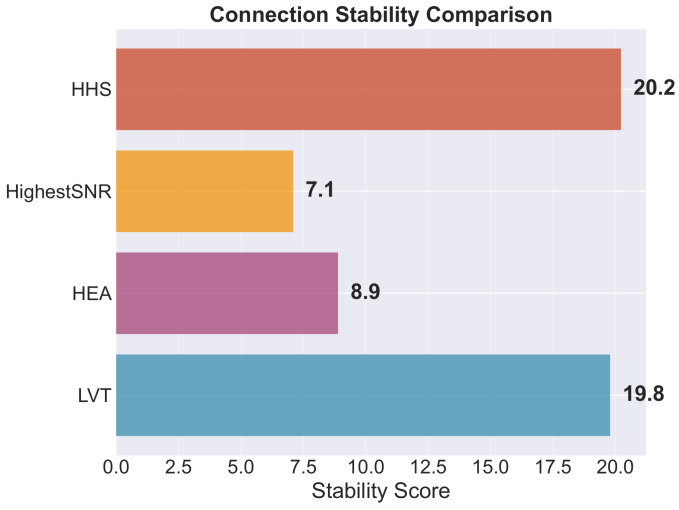
Connection stability score across algorithms, computed as the ratio of total simulation time to the number of handovers (higher is better). HHS achieves the highest stability score (20.2), indicating longer average connection durations between handovers.

Figure 10 illustrates the stability score metric. This metric serves as a composite measure of connection continuity. The score is determined by multiplying the average connection duration by the service availability. Finally, the result is normalized to a scale of 100. The mathematical representation of this metric is $S=(T_{\mathrm{avg}}\times A)/100$. In this formula, $T_{\mathrm{avg}}$ represents the mean connection duration in seconds, while *A* denotes the service availability percentage. The HHS algorithm achieves the highest stability score of 20.2. This performance marginally exceeds that of LVT, which records a score of 19.8. It is important to note that the HHS maintains this lead even though LVT exhibits lower overall availability. In contrast, quality-focused algorithms demonstrate substantially lower stability levels. Specifically, the Highest SINR and HEA algorithms record scores of 7.1 and 8.9, respectively. These results confirm that strategies prioritizing signal quality over the connection duration are less suitable for applications requiring high levels of network stability.

#### 6.2.2. Performance Trade-Offs

**Figure 11:**
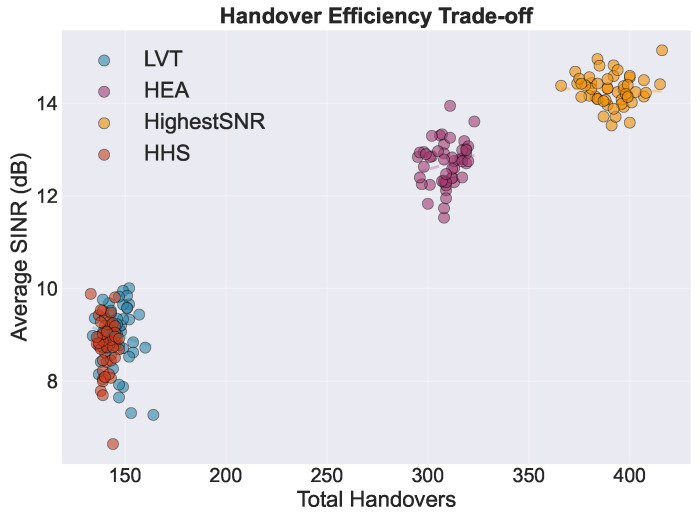
Handover efficiency trade-off showing average SINR versus total handover count for all Monte Carlo runs. Two distinct operational clusters are observed, i.e., a low-handover regime (HHS, LVT) and a high-SINR regime (HEA, Highest SINR), highlighting the inherent trade-off between link quality and switching frequency.

**Figure 12:**
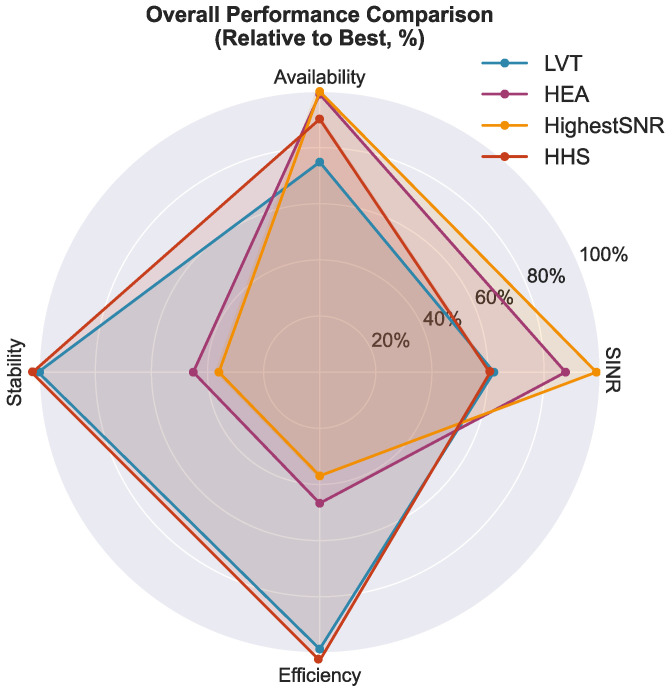
Multi-dimensional performance comparison using a radar chart with four normalized metrics: SINR, availability, stability, and efficiency (inverse of handover rate). Each metric is normalized relative to the best performer (100%), illustrating the overall performance balance achieved by the HHS across all dimensions.

Figure 11 presents the handover efficiency trade-off, plotting the average SINR against the total handovers for all simulation runs. The data reveal two distinct operational clusters: a high-efficiency regime occupied by the HHS and LVT (lower left) and a high-quality regime occupied by the HEA and Highest SINR algorithms (upper right). In terms of Pareto interpretation, the two-dimensional projection illustrates an approximate trade-off envelope. This envelope spans the low-handover region and the high-SINR region. It highlights that reductions in handover frequency necessarily come at the expense of link quality, and vice versa. In the low-handover cluster, the HHS and LVT achieve comparable positions in the handover–SINR plane. However, the HHS strictly dominates LVT when availability acts as a third performance dimension. Specifically, the HHS reaches 90.2% availability, while LVT reaches only 74.7%. This result is visualized in the radar chart in Figure 12. This three-dimensional dominance establishes the HHS as the preferred operating point for applications prioritizing connection stability without sacrificing service continuity. Overall, the HHS exhibits a robust balance between link quality and stability, with consistent performance across Monte Carlo realizations.

The multi-dimensional performance comparison in Figure 12 provides a holistic view of the algorithm performance across four normalized metrics: the SINR, availability, stability, and efficiency (inverse of handover rate). Each metric is normalized relative to the best performer (100%). The HHS demonstrates the most balanced performance profile, achieving high scores in stability (100%) and efficiency (96%) while maintaining acceptable levels for the SINR (61%) and availability (90%). In contrast, the Highest SINR algorithm excels in link quality (100%) and availability (100%) but performs poorly in stability (35%) and efficiency (37%). This visualization confirms that the HHS successfully navigates the multi-objective optimization space to achieve balanced performance. The observed trade-off between the handover rate and link quality aligns with the theoretical linearization presented in Section 5, where increasing the load weight $w_{\mathrm{load}}$ was predicted to reduce the handover frequency at the cost of the average SINR. The empirical reduction of 64% in handovers for a 5.5 dB SINR penalty validates the first-order approximation in Equations (57) and (58).

#### 6.2.3. Discussion of Limitations

While this study employs a high-fidelity simulation framework, it is important to acknowledge its methodological limitations, which also suggest avenues for future research. The assumption of a static ground terminal is a primary simplification; incorporating user mobility, particularly for high-speed aeronautical or maritime terminals, would introduce additional complexity to the handover problem [34,35]. Furthermore, our network model does not include intersatellite links (ISLs), which are a key feature of modern mega-constellations. The inclusion of ISLs would transform the problem from a simple link selection task to a more complex handover and routing co-design challenge [12].

The traffic model assumes that user arrivals follow a Poisson process, which is a common and analytically tractable choice in network simulation [27]. However, real-world Internet traffic exhibits burstiness, long-range dependence, and heavy-tailed session durations that the Poisson model does not capture. Under bursty traffic, satellite load fluctuations become more pronounced and temporally correlated. This behavior potentially increases the effective variability of the utility gap. Consequently, it affects the tightness of the derived probability bounds. Nevertheless, the relative ranking of handover strategies is expected to remain valid. The proposed stability mechanism and baselines rely primarily on geometry-driven and SINR-driven decision logic rather than fine-grained traffic arrival statistics. Furthermore, all algorithms are evaluated under identical traffic assumptions.

The channel model, while thorough, does not consider all possible impairments. Ionospheric scintillation, which may be substantial in specific geographic areas, is not explicitly modeled [36]. Additionally, residual Doppler estimation errors and imperfect compensation may further affect short-term SINR fluctuations. Incorporating self-similar traffic models and explicit Doppler impairments would provide a more rigorous stress test of the proposed algorithm.

These limitations suggest several concrete extension directions. For mobile ground terminals, the proposed framework can be extended. This would involve augmenting the utility function with mobility-aware predictors, such as the expected visibility duration or Doppler evolution. Consequently, this approach would enable proactive rather than purely reactive handover decisions. In the presence of ISLs, the HHS could be generalized from a link selection policy to a joint access–routing strategy. In this scenario, the utility gap would incorporate both the access link quality and downstream path cost over the ISL mesh. Such an extension would naturally couple handover decisions with dynamic routing metrics. At the same time, it would preserve the low-complexity, threshold-based structure of the proposed approach.

## 7. Conclusions

In this paper, we have proposed the HHS, a low-complexity algorithm for LEO satellite networks that balances connection stability with link quality through a multi-attribute utility function incorporating a novel logistic-decay stability bonus. The theoretical analysis established distribution-free bounds on the ping-pong probability and demonstrated $O(N_{v})$ computational complexity, significantly lower than that of existing machine learning approaches. Monte Carlo simulations using real Starlink TLE data validated that the HHS reduces the handover frequency by 64% compared to the Highest SINR strategy while maintaining 90.2% service availability, outperforming both aggressive quality-focused and conservative stability-focused benchmarks. The algorithm achieved the highest stability score (20.2) among all tested strategies, confirming its suitability for applications requiring stable connections. The proposed algorithm relies only on standard inputs (e.g., SINR measurements, satellite geometry, and broadcast load indicators). It requires no modifications to physical-layer procedures or signaling protocols. This makes it suitable for integration into existing satellite access systems. Future work will extend the framework to multi-terminal scenarios with intersatellite links, incorporate user mobility patterns for aeronautical and maritime applications, and investigate the integration of QoE metrics for multimedia services over LEO networks.

## Figures and Tables

**Table 1:** Comparative analysis of recent LEO handover strategies.

Ref.	Year	Core Algorithm	Decision Metrics	Load Model	Key Advantages	Main Limitations
[6]	2022	MADM (TOPSIS)	Delay, Load	User-defined	Reduces handovers and terminations	Weight sensitivity; no sensitivity analysis
[7]	2023	Utility (Greedy)	Backhaul, Service Time, Handover Factor	Backhaul (ISL)	Max. backhaul, low handover freq.	Not globally optimal
[9]	2022	Auction (SPA)	Signal, Service Time	None	Distributed, scalable, low overhead	Assumes rational agents
[12]	2020	Routing (SSLB)	N/A (Routing)	ISL bandwidth	Network-wide load balance	Low-latitude only
[14]	2023	DRL (DQN)	CNR, RBs, UE pos.	RBs	Eliminates measurement report	Training complexity
[15]	2024	DRL + GNN	Time, Delay, Rate	Via QoS	Dynamic topology adaptation	High complexity and training
[16]	2025	DRL + LSTM	Switching, Quality, Load	LSTM prediction	Proactive traffic-based	Joint prediction complexity

**Table 2:** Simulation configuration and algorithm parameters.

Parameter Category	Value
System Architecture	
Constellation	Starlink (filtered set)
TLE Source	CelesTrak, SGP4 via Skyfield
Satellite Selection Criteria	Mean motion: 14.9–15.2 rev/day, |β*|<0.0001, TLE age <30 days
Ground Terminal Location	Riyadh, SA (24.7° N, 46.7° E)
Downlink Frequency (*f*)	10.75 GHz (Ku band)
Bandwidth (*B*)	250 MHz
Noise Figure (NF)	0.8 dB
Minimum Elevation ($\theta_{\min}$)	5.0°
Simulation Framework	
Monte Carlo Runs	50 per algorithm (200 total)
Simulation Duration	2 h (7200 s) per run
Decision Epoch (Δt)	10 s
Start-Time Distribution	Randomized within 7-day window
Computational Architecture	Parallel execution via joblib library
Statistical Independence	Independent seeding per run
Channel and Traffic Model	
Channel Impairments	FSPL, ITU-R P.676, ITU-R P.838, ITU-R P.618
Interference	Co-channel aggregate
Traffic Grid Resolution (G)	1.0°
Internet Penetration ($P_{\mathrm{pen}}$)	0.01–0.85 (region-dependent)
Diurnal Multipliers	$m_{\mathrm{night}}=0.3$, $m_{\mathrm{day}}=0.7$, $m_{\mathrm{eve}}=1.0$
Baseline Algorithms	
LVT Time-to-Trigger	240 ms, Hysteresis: 3.0 dB
HEA Hysteresis	4.0°
Highest SINR Hysteresis	2.0 dB
HHS Parameters	
Quality Weights	$w_{\gamma }=0.5$, $w_{\theta }=0.15$, $w_{\mathrm{load}}=0.25$
Stability Weight	$w_{\mathrm{stab}}=0.1$
Handover Penalty ($P_{\mathrm{ho}}$)	0.03
Opportunistic Margin ($\Delta U_{\mathrm{th}}$)	0.02
Degradation Threshold	$\gamma_{\mathrm{th}}=8.0$ dB
Stability Parameters	κ=0.2, τ=15 s

**Table 3:** Performance comparison of LEO handover algorithms (mean ± std. dev. from 50 Monte Carlo runs).

Algorithm	Handovers	SINR (dB)	Availability (%)	Stability Score
HHS (Proposed)	140.9±3.1	8.8±0.6	90.2±1.8	20.2
Highest SINR	390.9±10.9	14.3±0.3	99.8±0.3	7.1
HEA	309.2±7.4	12.7±0.5	98.9±0.9	8.9
LVT	146.4±6.1	9.0±0.6	74.7±2.5	19.8

## Data Availability

The two-line element (TLE) data for the Starlink constellation are publicly available from CelesTrak (https://celestrak.org/).

## Abbreviations

The following abbreviations are used in this manuscript:

3GPP	3rd-Generation Partnership Project
AI	Artificial Intelligence
ANOVA	Analysis of Variance
CNR	Carrier-to-Noise Ratio
DHO	Deep Handover
DQN	Deep Q-Network
DRL	Deep Reinforcement Learning
ECEF	Earth-Centered, Earth-Fixed
EIRP	Effective Isotropic Radiated Power
FL	Fuzzy Logic
FSPL	Free-Space Path Loss
GNN	Graph Neural Network
GT	Ground Terminal
HEA	Highest Elevation Angle
HHS	Hybrid Handover Strategy
IoT	Internet of Things
IQR	Interquartile Range
ISL	Intersatellite Link
ITU	International Telecommunication Union
KPI	Key Performance Indicator
LEO	Low Earth Orbit
LoS	Line-of-Sight
LSTM	Long Short-Term Memory
LVT	Longest Visibility Time
MADM	Multi-Attribute Decision Making
MCDM	Multi-Criteria Decision Making
ML	Machine Learning
NTN	Non-Terrestrial Network
QoE	Quality of Experience
QoS	Quality of Service
RB	Resource Block
RL	Reinforcement Learning
RSRP	Reference Signal Received Power
RSSI	Received Signal Strength Indicator
SGP4	Simplified General Perturbations 4
SINR	Signal-to-Interference-plus-Noise Ratio
SNR	Signal-to-Noise Ratio
SPA	Second Price Auction
SSLB	Selective Split Load Balancing
TLE	Two-Line Element
TN	Terrestrial Network
TOPSIS	Technique for Order Preference by Similarity to Ideal Solution
TTT	Time-to-Trigger
UE	User Equipment
